# Development and Validation of an Anoikis-Related Machine Learning Signature for Prognosis and Brain Metastasis-Associated Classification in Lung Adenocarcinoma

**DOI:** 10.3390/cancers18121969

**Published:** 2026-06-17

**Authors:** Junhong Wu, Baijun Zhang, Hengrui Liu

**Affiliations:** Hong Kong Xieze Institute of Precision Medicine, Hong Kong 999077, China

**Keywords:** lung adenocarcinoma, brain metastasis, anoikis resistance, large language model, machine learning

## Abstract

**Background**: Brain metastasis is associated with poor prognosis in lung adenocarcinoma (LUAD). Anoikis resistance may contribute to tumor cell survival during metastatic dissemination and brain colonization; however, robust biomarkers for prognostic stratification and brain metastasis-associated classification remain limited. This study aimed to investigate anoikis-related molecular features in LUAD brain metastasis and develop a machine learning-based signature for prognostic assessment and exploratory classification of primary and brain-metastatic LUAD samples. **Methods**: We integrated single-cell and multi-cohort bulk transcriptomic data. Single-cell analysis was performed to characterize anoikis-related cellular states and intercellular communication in primary and brain-metastatic LUAD samples. In the bulk transcriptomic analysis, TCGA-LUAD was used for prognostic feature selection and risk-model construction, and GSE26939 was used for external prognostic validation. The classification performance of the fixed signature for distinguishing primary LUAD from brain-metastatic LUAD samples was further evaluated in GSE161116 and GSE271259. Immune microenvironment features were assessed, and an LLM-assisted exploratory drug-screening strategy combined with molecular docking was used to prioritize candidate compounds. **Results**: Single-cell analysis suggested that metastatic epithelial cells exhibited enhanced anoikis-related activity, accompanied by macrophage-associated SPP1-CD44 and MIF-(CD74+CXCR4) communication patterns. Machine learning-based feature selection identified an eight-gene signature consisting of *BIRC3*, *CCL20*, *CLEC7A*, *CTSL*, *GOLM1*, *ICAM3*, *MTUS1*, and *SERPINH1*. The signature showed prognostic value in TCGA-LUAD and GSE26939 and demonstrated exploratory classification performance in distinguishing primary LUAD from brain-metastatic LUAD samples. High-risk patients exhibited immune microenvironment alterations and enrichment of tumor progression-related pathways. LLM-assisted compound prioritization and molecular docking highlighted resveratrol and SB431542 as hypothesis-generating candidates with predicted interactions with core targets. **Conclusions**: This study identified an anoikis-related eight-gene signature for LUAD prognostic stratification and exploratory brain metastasis-associated classification. The findings suggest the potential involvement of anoikis-related tumor–microenvironment interactions in LUAD brain metastasis and provide candidate genes and compounds for further experimental validation.

## Simple Summary

Brain metastasis is a major cause of poor outcomes in patients with lung adenocarcinoma (LUAD). Tumor cells that spread to the brain must survive detachment from their original environment, a process that may be facilitated by anoikis resistance. In this study, we integrated single-cell and bulk transcriptomic datasets to investigate anoikis-related molecular features associated with LUAD brain metastasis. We identified an eight-gene signature that was associated with patient prognosis and showed exploratory ability to distinguish primary LUAD from brain-metastatic LUAD samples. Single-cell analyses suggested that macrophage-associated signaling pathways and tumor microenvironment interactions may be involved in metastatic adaptation. We further explored candidate compounds using an LLM-assisted prioritization strategy combined with molecular docking and performed preliminary experimental validation focusing on BIRC3 and SB431542. Although additional biological and clinical validation is required, our findings provide new insights into the molecular characteristics of LUAD brain metastasis and highlight candidate genes and compounds for future investigation.

## 1. Introduction

Lung adenocarcinoma (LUAD) is a prevalent malignancy worldwide [1,2,3,4], and brain metastasis (BM) is a critical factor contributing to its poor prognosis [5]. Approximately 40–50% of LUAD patients develop BM throughout the disease course, and survival remains limited once BM occurs [6,7]. Although targeted therapies against driver alterations such as EGFR and ALK, along with immunotherapy [8,9], have achieved remarkable progress in controlling systemic disease, the central nervous system can serve as a sanctuary site partly because of the presence of the blood–brain barrier (BBB), resulting in limited effective treatment options for patients with BM [10,11,12].

In the metastatic cascade, anoikis, a form of programmed cell death triggered upon detachment from the extracellular matrix, constitutes a critical barrier restricting distant dissemination [13]. Tumor cells with anoikis-resistant phenotypes may acquire survival advantages during circulation, BBB traversal, and brain colonization [14]. Previous studies have implicated epithelial–mesenchymal transition (EMT), aberrant receptor tyrosine kinase activation, and tumor microenvironment remodeling in anoikis resistance and LUAD progression [15,16,17]. Notably, MET amplification is significantly more frequent in brain metastases than in primary tumors and is strongly associated with extremely poor overall survival, suggesting that anoikis-related molecular features may provide insights into BM-associated biology and prognostic stratification [18]. However, existing transcriptomic studies are often limited by single-cohort designs, relatively small sample sizes, or insufficient cross-cohort validation. Moreover, biomarkers that can support prognostic assessment and exploratory classification of primary versus brain-metastatic LUAD samples remain insufficiently characterized.

In recent years, large language models (LLMs), exemplified by GPT-4, have shown potential for biomedical text interpretation, knowledge integration, and structured information extraction [19,20,21]. To address the aforementioned methodological bottlenecks, this study applied an LLM-assisted integrative machine learning framework (LLM-IMLF) [22,23] to support single-cell annotation, candidate-gene prioritization, and compound-prioritization analysis [24,25], and compared 101 machine learning algorithm combinations for prognostic signature construction. In this study, we integrated single-cell transcriptomic data from LUAD primary tumors and brain metastases to characterize anoikis-related cellular states and tumor–microenvironment communication patterns at single-cell resolution. Using multi-cohort bulk transcriptomic datasets, we identified an 8-gene anoikis signature that demonstrated excellent C-index and AUC values across multiple external validation cohorts, achieving comprehensive coverage from survival prediction to BM diagnosis, and revealing intrinsic associations between high-risk signatures and immune evasion phenotypes such as T-cell exhaustion. Finally, by combining LLM-driven intelligent screening with AlphaFold-based [26] structural simulations, we nominated candidate compounds for further validation.

Collectively, this study provides an anoikis-related machine learning signature for LUAD prognostic stratification and exploratory BM-associated classification and offers hypothesis-generating insights into tumor–microenvironment interactions and candidate compound prioritization in LUAD brain metastasis.

## 2. Materials and Methods

### 2.1. Data Acquisition and Preprocessing

Multi-dimensional transcriptomic data of lung adenocarcinoma (LUAD) were obtained from public databases. The single-cell RNA-seq datasets GSE131907 and GSE148071, including 8 primary LUAD tissues and 11 brain-metastatic LUAD tissues, were used to characterize the metastatic microenvironment. For bulk transcriptomic analysis, TCGA-LUAD was used as the primary training cohort for prognostic feature selection and risk-model construction, and GSE26939 was used as an independent prognostic validation cohort (Supplementary Table S2). GSE161116 and GSE271259 were used to evaluate the classification performance of the fixed eight-gene signature in distinguishing primary LUAD from brain-metastatic LUAD samples (Supplementary Table S3). Before downstream analysis, gene symbols were unified across datasets, duplicated gene symbols were collapsed by retaining the probe or transcript with the highest average expression, and expression matrices were log2-transformed when necessary according to the data distribution and platform annotation.

### 2.2. Single-Cell Data Processing and LLM-Assisted Cell Annotation

The raw count matrix was processed using the R package Seurat (v5.0.1). Cells were retained according to the following quality-control criteria: 500–6000 detected genes per cell, a mitochondrial gene proportion <20%, and total UMI counts >1000. After quality control, the data were normalized using NormalizeData(), and the top 2000 highly variable genes were identified using FindVariableFeatures(). Principal component analysis (PCA) was performed based on the highly variable genes, and the first 13 principal components were retained for downstream analysis. Cell neighbors were constructed using FindNeighbors() based on the selected 13 PCs, followed by unsupervised clustering using FindClusters() with a resolution of 0.8. UMAP dimensionality reduction was performed using RunUMAP() with the same 13 PCs for visualization. To assist cell-type annotation, cluster-level marker-gene summaries were used as input for LLM-assisted annotation, and each model was prompted to assign one predefined cell-type label with marker-based supporting evidence. Multiple annotation methods, including GPT-4, Claude 3, Gemini 1.5, Llama 3, scGPT (v0.2.1), CellTypist (v1.7.1), SingleCellNet (v0.1.0), and SingleR (v2.10.0), were compared against marker-based reference annotations. Standardized outputs were evaluated using confusion matrices, accuracy, precision, and weighted F1-scores at the cluster level. Detailed prompts, model settings, output schema, and repeated-run strategy are provided in Supplementary Table S4, and benchmarking results and confusion matrices are provided in Supplementary Tables S5 and S6.

### 2.3. Anoikis Scoring and Cell–Cell Communication Analysis

Anoikis scores were calculated at the single-cell level using the AUCell algorithm (v1.24.0) to evaluate anoikis-related activity across different cell populations [27]. Differences in scores between primary and metastatic lesions were assessed using the Wilcoxon test.

Cell–cell communication analysis was performed using CellChat (v2.1.2) with the human CellChatDB reference. Communication probabilities were inferred from normalized expression data and annotated cell identities, followed by pathway-level aggregation and centrality analysis to evaluate sender, receiver, mediator, and influencer roles. Low-confidence interactions were filtered according to the default CellChat workflow, and the SPP1 and MIF signaling pathways were further examined. These analyses were interpreted as inferred intercellular communication patterns rather than direct evidence of causal signaling.

### 2.4. Differential Expression Analysis and Candidate Gene Screening

Differentially expressed genes (DEGs) between primary and brain-metastatic lesions were identified using the FindMarkers() function in Seurat with the Wilcoxon rank-sum test. Genes with an adjusted *p* < 0.05 and |log_2_ fold change| > 0.25 were considered differentially expressed. Candidate anoikis-related genes were obtained by intersecting DEGs with a curated anoikis-related gene list from published sources (Supplementary Table S7). In the TCGA-LUAD training cohort, univariate Cox regression analysis was performed to identify candidate genes significantly associated with overall survival. Genes with *p* < 0.05 were retained for subsequent machine learning-based feature selection.

### 2.5. Machine Learning-Based Prognostic Signature Construction

To identify a robust prognostic signature, machine learning-based feature selection was performed strictly within the TCGA-LUAD training cohort. A total of 101 machine learning algorithm combinations were compared for prognostic model construction, and model performance was evaluated using the concordance index (C-index). The performance matrix of the 101 algorithm combinations is provided in Supplementary Table S8. Feature dimensionality reduction was conducted using least absolute shrinkage and selection operator (LASSO) regression with the glmnet package and random survival forest (RSF) with the randomForestSRC package. Genes with non-zero LASSO coefficients and high RSF variable importance were prioritized, and eight core genes (*BIRC3*, *CCL20*, *CLEC7A*, *CTSL*, *GOLM1*, *ICAM3*, *MTUS1*, and *SERPINH1*) were selected to construct the final signature. The risk score for each patient was calculated as the sum of the expression levels of each gene weighted by their corresponding Cox regression coefficients. The final risk-score formula was locked after training in TCGA-LUAD. External cohorts were not used for feature selection, coefficient estimation, cutoff determination, or prognostic model refitting.

### 2.6. Prognostic Validation and Classification Evaluation

For prognostic validation, the locked eight-gene risk-score formula derived from TCGA-LUAD was applied to both the TCGA training cohort and the external GSE26939 cohort. Patients were divided into high- and low-risk groups using the median risk score derived from the TCGA-LUAD training cohort, and the same cutoff was directly applied to GSE26939 without re-estimation. Survival differences between groups were assessed using Kaplan–Meier survival curves and the log-rank test. Time-dependent ROC curves at 1, 3, and 5 years were calculated using the timeROC package. For exploratory clinical assessment, a nomogram integrating the risk score with clinical stage was constructed using the rms package, and the agreement between predicted survival rates and actual observations was evaluated using calibration curves.

### 2.7. Immune Microenvironment Assessment and Functional Enrichment Analysis

The CIBERSORT algorithm with the LM22 signature matrix was used to estimate the relative abundances of 22 immune cell types in the TCGA cohort. Spearman correlation analysis was performed to evaluate associations between the risk score and the expression levels of immune checkpoint genes, including *TIGIT*, *PDCD1*, *CTLA4*, *CD244*, *HAVCR2*, and *CD160*. Gene set enrichment analysis (GSEA) was conducted using clusterProfiler on the Hallmark gene sets from MSigDB, with 1000 permutations. Pathways with |NES| > 1 and adjusted *p* < 0.05 were considered significantly enriched. Immune-related findings were interpreted as bulk transcriptome-derived associations rather than direct evidence of functional immune states.

### 2.8. Drug Screening and Molecular Docking Validation

Candidate small molecules targeting the eight core genes were retrieved from the Comparative Toxicogenomics Database (CTD). An LLM-based intelligent screening protocol was then constructed to evaluate candidates based on pharmacological activity, toxicity, and potential blood–brain barrier (BBB) penetrability. For structural validation, three-dimensional models of the eight target proteins were obtained from the AlphaFold Database. Ligands in SDF format were converted to PDBQT using Open Babel, and molecular docking was performed using AutoDock Vina (v1.2.7) with Exhaustiveness set to 12. Binding affinities (kcal/mol) for each drug–target pair were recorded and visualized as a heatmap.

### 2.9. Cell-Based Suspension Model and qRT-PCR Validation

To model matrix-detachment stress relevant to anoikis, commercially sourced human LUAD PC-9 cells were cultured under adherent conditions or suspension conditions. Adherent cells grown on standard tissue-culture plates served as controls, whereas suspension-model cells were maintained on ultra-low-attachment plates for 24 h. Total RNA was extracted using the Total RNA Isolation Kit (G3013, Servicebio, Wuhan, China). cDNA was synthesized, and qRT-PCR was performed to quantify BIRC3, CCL20, and MMP9. Relative expression levels were normalized to GAPDH and calculated using the 2^−ΔΔCt^ method. Primer sequences used for qRT-PCR are listed in Supplementary Table S9.

### 2.10. Cell Culture and SB431542 Treatment

Human LUAD PC-9 cells were used for the in vitro validation experiments. PC-9 is a human lung adenocarcinoma cell line recorded in Cellosaurus (CVCL_B260). The cell line identity was confirmed by STR profiling, which showed a 93.62% match with the reference PC-9 profile. Cells were verified to be free of mycoplasma contamination and used within passages 3 to 12. The cells were cultured in RPMI-1640 medium supplemented with 10% fetal bovine serum and 1% penicillin–streptomycin at 37 degrees Celsius in a humidified atmosphere with 5% CO_2_. For downstream functional and molecular assays, cells were treated with specific concentrations of SB431542 (S1067, Selleck, Houston, TX, USA) dissolved in DMSO for 24 h. Vehicle-control cells received an equivalent volume of DMSO, with the final concentration strictly maintained below 0.1% (*v/v*).

### 2.11. Western Blotting

To evaluate the dose-dependent effects of SB431542, non-transfected LUAD cells were treated with vehicle, 5 uM, or 10 uM SB431542 for 24 h. Total protein was extracted using RIPA Lysis Buffer (G2002, Servicebio, Wuhan, China) supplemented with protease and phosphatase inhibitors. Equal amounts of protein (30 ug per lane) were separated by SDS-PAGE using a rapid gel preparation kit (G2037-50T, Servicebio, Wuhan, China) and transferred onto PVDF membranes. A prestained protein marker (G2089, Servicebio, Wuhan, China) was used to estimate molecular weights. After blocking, membranes were incubated with primary antibodies against BIRC3 (GB11511, 1:1000, Servicebio, Wuhan, China), cleaved caspase-3 (GB11532, 1:1000, Servicebio, Wuhan, China), MMP9 (GB11132, 1:1000, Servicebio, Wuhan, China), and beta-actin (GB12001, 1:3000, Servicebio, Wuhan, China) at 4 degrees Celsius overnight. Membranes were then incubated with the corresponding secondary antibodies diluted in Western Secondary Antibody Dilution Buffer (G2009, Servicebio, Wuhan, China). Protein bands were visualized using an enhanced chemiluminescence detection system. Target band intensities were quantified using ImageJ software (v1.54), and relative protein levels were determined by direct normalization to beta-actin as the loading control. All Western blotting experiments were performed in triplicate (*n* = 3).

### 2.12. BIRC3 Overexpression

To perform rescue assays, LUAD cells were transfected with a BIRC3-overexpression plasmid (BIRC3-OE) or the corresponding empty-vector negative control (oe-NC) using Lipofectamine 3000 transfection reagent (Invitrogen, Carlsbad, CA, USA) according to the manufacturer’s protocol. After 48 h of transfection, cells were collected and prepared for downstream pharmacological treatments and invasion assays. To assess whether BIRC3 was associated with SB431542-related changes in invasive capacity, cells were treated with vehicle or high-dose (10 uM) SB431542 and assigned to four definitive experimental groups: control, SB431542 alone, SB431542 + oe-NC, and SB431542 + BIRC3-OE.

### 2.13. Transwell Invasion Assay

Cell invasion was assessed using 24-well Transwell inserts with 8 um pores pre-coated with Matrigel (BD Biosciences, San Jose, CA, USA). A total of 5 × 10^4^ cells suspended in 200 uL of serum-free medium were seeded into the upper chamber. For groups involving pharmacological treatment, 10 uM SB431542 or the matching vehicle control (DMSO) was maintained in both the upper and lower chambers. Complete medium containing 10% FBS (600 uL) was added to the lower chamber as a chemoattractant. After a 24 h incubation period, non-invading cells remaining on the upper surface of the membrane were carefully removed with a cotton swab. The invaded cells on the lower surface were fixed with 4% paraformaldehyde, stained with 0.1% crystal violet, and photographed at 100× magnification under a light microscope. To ensure objectivity, the number of invaded cells was quantified in five randomly selected fields per insert by investigators who were completely blinded to the experimental group allocation.

### 2.14. Statistical Analysis of Experimental Data

Experimental data are presented as the mean ± SD from three independent experiments. Student’s *t*-test was used for two-group comparisons, and one-way analysis of variance followed by Tukey’s post hoc test was used for comparisons among multiple groups. A two-sided *p* < 0.05 was considered statistically significant.

## 3. Results

### 3.1. LLM-Assisted High-Precision Single-Cell Atlas Construction and Anoikis Assessment

**Figure 1:**
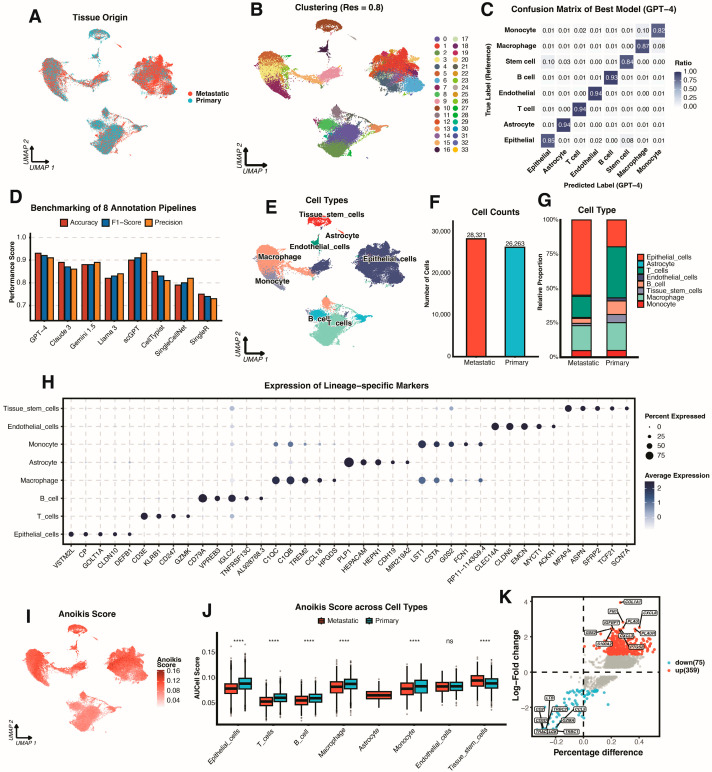
Single-cell atlas of LUAD and evaluation of LLM-based annotation. (**A**) UMAP colored by tissue origin. (**B**) UMAP of 34 unsupervised clusters. (**C**) Confusion matrix of GPT-4 annotation. (**D**) Accuracy, F1-score, and precision across eight annotation tools. (**E**) UMAP with annotated cell types. (**F**,**G**) Total cell counts (**F**) and cell type proportions (**G**) across groups. (**H**) Dot plot of lineage-specific marker genes. (**I**) UMAP of anoikis score distribution. (**J**) Boxplot of anoikis scores across cell types between primary and metastatic lesions (**** *p* < 0.0001). (**K**) Scatter plot of differentially expressed genes; red: upregulated (*n* = 359), blue: downregulated (*n* = 75).

Integrated single-cell transcriptomic analysis of primary and brain-metastatic lesions was performed. Unsupervised clustering of 54,584 high-quality cells revealed distinct transcriptional clusters (Figure 1A,B). Benchmarking of eight annotation tools showed that GPT-4 achieved the highest overall performance, including the highest accuracy and weighted F1-score in identifying key lineages (epithelial cells, macrophages, monocytes), outperforming Claude 3, Gemini 1.5, and SingleR (Supplementary Table S1; Figure 1C,D). Major cell lineages (epithelial cells, T cells, B cells, and macrophages, astrocytes) were defined (Figure 1E–G), with marker-gene expression supporting lineage specificity (Figure 1H).

Anoikis scores calculated by AUCell revealed that epithelial cells in brain-metastatic lesions exhibited significantly elevated anoikis-related activity (Figure 1I). Epithelial cells, macrophages, and tissue stem cells showed significantly higher anoikis scores in metastases than in primary lesions (Figure 1J). Differential expression analysis identified 359 upregulated and 75 downregulated genes between primary and brain-metastatic lesions (Figure 1K).

### 3.2. Cell–Cell Communication Landscape in the Metastatic Microenvironment

**Figure 2:**
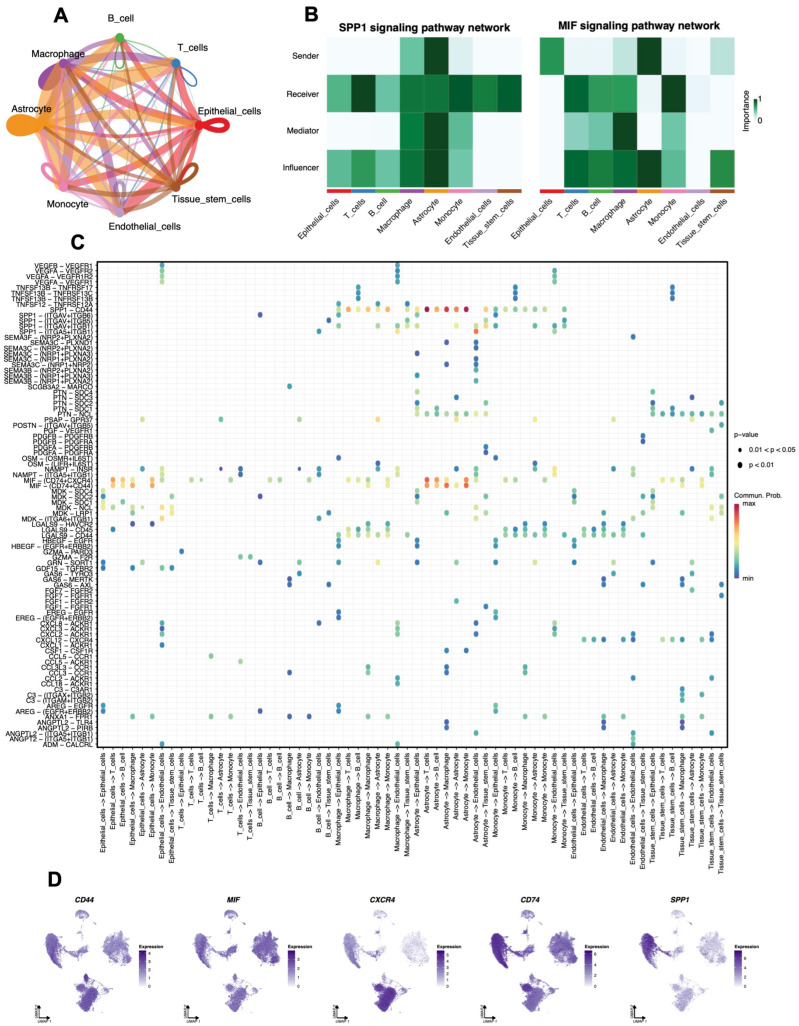
Cell–cell communication networks and core signaling pathways. (**A**) Network diagram of interaction intensities among cell types; line thickness indicates communication strength. (**B**) Heatmap of cell roles in the *SPP1* and *MIF* pathways; color intensity represents importance scores as senders, receivers, mediators, and influencers. (**C**) Bubble plot of ligand–receptor communication probabilities across cell pairs; color indicates probability, size indicates *p*-value. (**D**) UMAP plots showing expression of core communication genes (*CD44*, *MIF*, *CXCR4*, *CD74*, and *SPP1*).

To investigate intercellular communication in the brain-metastatic microenvironment, we constructed an interaction network among major cell lineages (Figure 2A). The SPP1 and MIF signaling pathways predominated in cell–cell communication. Heatmap analysis revealed that epithelial cells and macrophages served as key senders and receivers in these pathways (Figure 2B). Analysis of specific ligand–receptor pairs identified high communication probabilities for the *MIF*-(*CD74*+*CXCR4*), *MIF*-(*CD74*+*CD44*), and *SPP1*-*CD44* axes across multiple cell pairs (Figure 2C). Feature plot validation confirmed that these key ligand and receptor genes were highly expressed in brain-metastatic lesion-associated cell clusters (Figure 2D), suggesting that these signaling axes may be associated with tumor–microenvironment communication patterns in the brain-metastatic microenvironment.

### 3.3. Construction of an Anoikis-Related Prognostic Signature and Exploratory Classification Assessment

**Figure 3:**
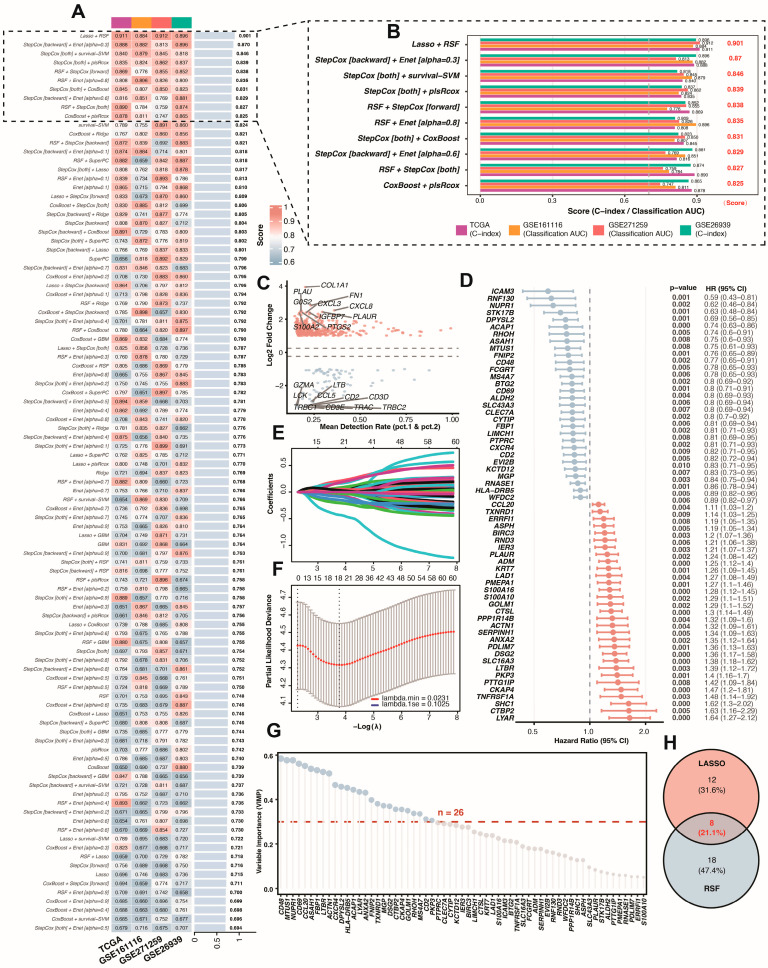
Feature screening workflow for prognostic signature construction and exploratory classification assessment. (**A**) Heatmap of 101 algorithm combinations across prognostic and exploratory classification cohorts. (**B**) Top 10 prognostic models ranked by C-index performance; the optimal model (Lasso + RSF) in shown in red. (**C**) Differential expression distribution of candidate genes. (**D**) Forest plot of prognostic candidate genes from univariate Cox regression. (**E**) LASSO regression: coefficient paths. (**F**) Ten-fold cross-validation curve for LASSO regression. (**G**) Variable importance (VIMP) ranking from RSF. (**H**) Venn diagram showing the intersection of LASSO and RSF.

To develop a robust prognostic model, we constructed an ensemble framework comprising 101 machine learning algorithm combinations. Comparison of 101 machine learning algorithm combinations within the TCGA-LUAD training cohort identified the “Lasso + RSF” combination as the optimal prognostic model based on C-index performance (Supplementary Table S8; Figure 3A,B). The fixed signature was subsequently evaluated in external prognostic and classification cohorts. For feature screening, candidate genes with significant prognostic value were identified by integrating single-cell differential expression analysis (Figure 3C) with univariate Cox regression (Figure 3D). Dimensionality reduction was further performed using LASSO regression (Figure 3E,F) and random survival forest (RSF) (Figure 3G). Taking the intersection of genes selected by both algorithms ultimately identified eight core anoikis-related genes: *BIRC3*, *CCL20*, *CLEC7A*, *CTSL*, *GOLM1*, *ICAM3*, *MTUS1*, and *SERPINH1* (Figure 3H).

### 3.4. Prognostic Validation of the Anoikis Signature in LUAD

**Figure 4:**
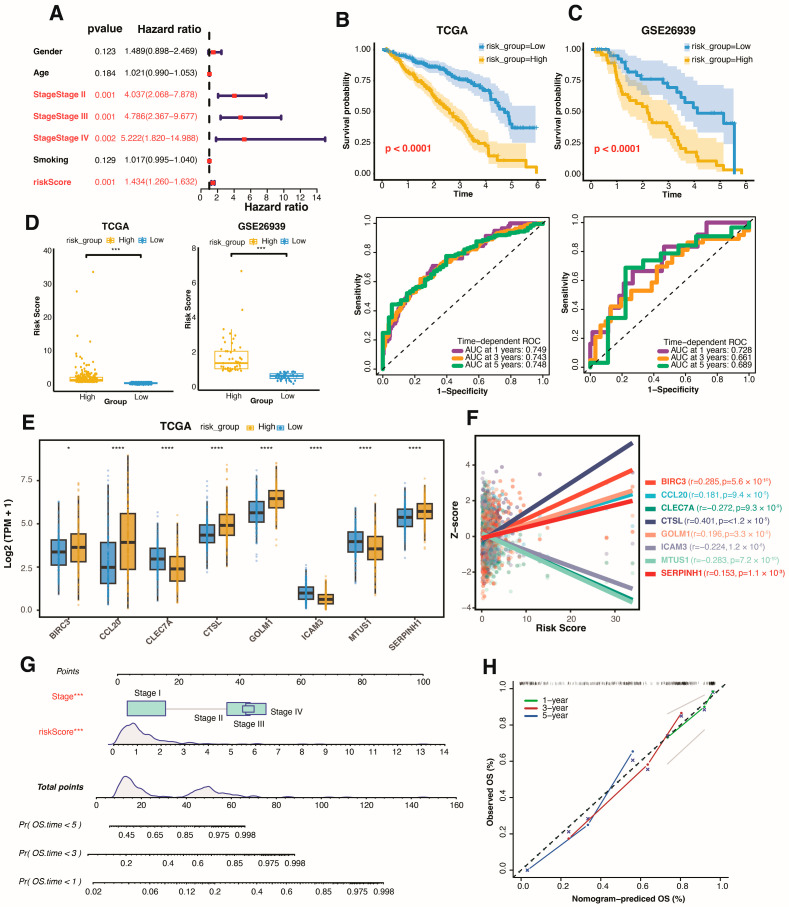
Prognostic performance and clinical application of the signature model. (**A**) Forest plot of independent prognostic analysis. (**B**,**C**) Kaplan–Meier survival curves (**top**) and time-dependent ROC curves (**bottom**) for TCGA (**B**) and GSE26939 (**C**) cohorts. (**D**) Boxplot of risk score distribution between high and low risk groups. (**E**) Boxplot of the expression levels of eight core genes between risk groups. (**F**) Scatter plots of correlations between gene expression and risk score. (**G**) Nomogram for predicting 1-, 3-, and 5-year survival. (**H**) Calibration curves for survival prediction. * *p* < 0.05, *** *p* < 0.001, **** *p* < 0.0001.

A prognostic risk score model was constructed using multivariate Cox regression based on the eight core anoikis-related genes. Regression coefficients and hazard ratios (HRs) for each gene are presented in Table 1. *BIRC3*, *CCL20*, *CTSL*, *GOLM1*, and *SERPINH1* were identified as risk factors (HR > 1), whereas *CLEC7A*, *ICAM3*, and *MTUS1* were protective factors (HR < 1). Cox regression analysis confirmed that the risk score was an independent prognostic factor for LUAD patients (Figure 4A). In both the TCGA and GSE26939 cohorts, patients in the high-risk group exhibited significantly shorter survival times compared to the low-risk group (Figure 4B,C, *p* < 0.0001). Time-dependent ROC curves demonstrated high AUC values for 1-, 3-, and 5-year survival predictions (Figure 4B,C). Risk scores were significantly higher in the high-risk group (Figure 4D), and the core genes showed distinct expression patterns between risk groups (Figure 4E), with expression levels strongly correlated with the final risk score (Figure 4F). For clinical application, a nomogram integrating risk score and clinical stage was constructed (Figure 4G), and calibration curves showed agreement between predicted and observed survival rates (Figure 4H).

### 3.5. Immune Microenvironment Characteristics and Pathway Enrichment Analysis

**Figure 5:**
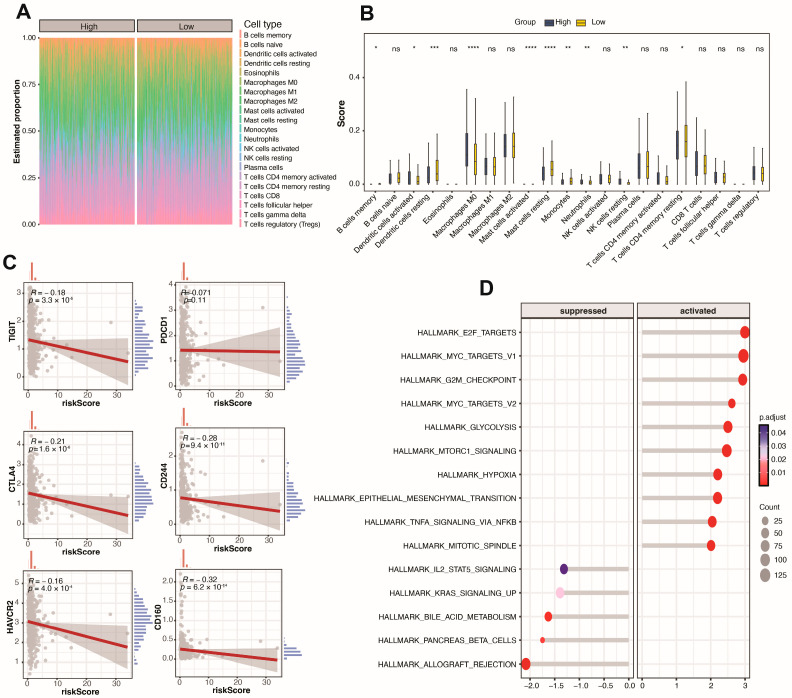
Immune microenvironment characteristics and GSEA pathway analysis. (**A**) Stacked bar plot of the proportions of 22 immune cell types between high and low risk groups. (**B**) Boxplot comparing immune cell infiltration scores. (**C**) Scatter plots of correlations between risk score and immune checkpoint gene expression. (**D**) GSEA enrichment analysis; activated pathways are shown on the right, and suppressed pathways are shown on the left. * *p* < 0.05, ** *p* < 0.01, *** *p* < 0.001, **** *p* < 0.0001.

CIBERSORT analysis revealed significant heterogeneity in immune cell infiltration between the high- and low-risk groups (Figure 5A,B). Correlation analysis showed that the risk score was significantly negatively correlated with the expression levels of core immune checkpoint genes, including *TIGIT*, *CTLA4*, *CD244*, *HAVCR2*, and *CD160* (Figure 5C), suggesting that high-risk scores may be associated with an immunosuppressive state. GSEA revealed that the high-risk group exhibited significant enrichment of pro-tumor pathways, including *E2F* targets, *MYC* targets, G2M checkpoint, and epithelial–mesenchymal transition (EMT) (Figure 5D), suggesting potential biological features associated with poor prognosis in the high-risk group.

### 3.6. LLM-IMLF-Based Candidate Compound Prioritization and Molecular Docking Assessment

**Figure 6:**
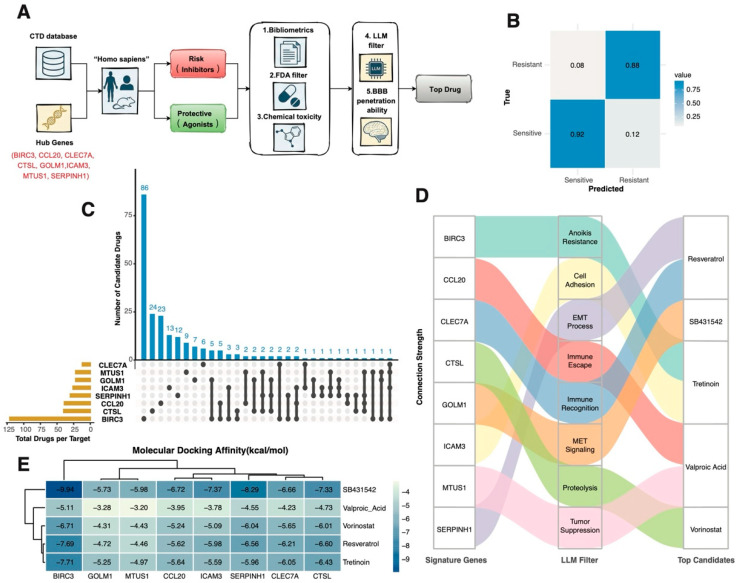
LLM-assisted candidate compound prioritization and molecular docking assessment. (**A**) Flowchart of the screening protocol with five filtering steps. (**B**) Confusion matrix of drug sensitivity prediction. (**C**) UpSet plot of candidate drug distribution across eight targets. (**D**) Sankey diagram linking signature genes to top drugs via biological processes. (**E**) Heatmap of binding affinities between five drugs and eight targets.

To prioritize candidate compounds associated with the eight-gene signature, we constructed an LLM-assisted screening protocol (Figure 6A). This protocol integrated species filtering, functional relevance assessment, pharmacological evidence review, and predicted blood–brain barrier (BBB)-related features to identify candidate molecules for further evaluation. The prediction performance of the screening framework was summarized using a confusion matrix (Figure 6B). The UpSet plot showed the distribution of 231 candidate compounds across the eight targets, with BIRC3 associated with the largest number of candidate compounds (Figure 6C). The Sankey diagram illustrated the relationships among signature genes, biological functions, and prioritized compounds, highlighting five representative candidates: resveratrol, SB431542, tretinoin, valproic acid, and vorinostat (Figure 6D). Batch molecular docking predicted favorable binding affinities between these five compounds and the eight target proteins, with SB431542 showing a binding energy of −9.94 kcal/mol for *BIRC3* (Figure 6E). These findings provide preliminary, hypothesis-generating evidence for candidate compound prioritization in LUAD brain metastasis and require further pharmacokinetic and functional validation.

### 3.7. Experimental Validation of BIRC3-Associated Invasive Phenotypes

**Figure 7:**
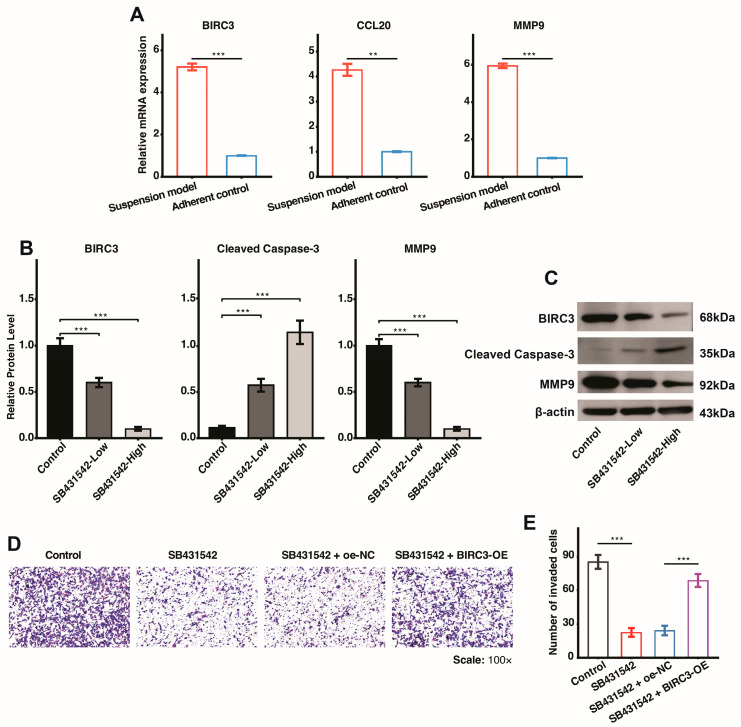
Experimental validation of *BIRC3*-associated invasion and SB431542 response. (**A**) qRT-PCR analysis of *BIRC3*, *CCL20*, and *MMP9* expression in adherent control and suspension model. (**B**) Quantification of *BIRC3*, *cleaved caspase-3*, and *MMP9* protein levels in LUAD cells treated with control, low-dose SB431542, or high-dose SB431542. (**C**) Representative Western blot images. The uncropped blots are shown in Supplementary Materials. (**D**) Representative Transwell invasion images of LUAD cells in the control, SB431542, SB431542 + oe-NC, and SB431542 + *BIRC3*-OE groups. (**E**) Quantification of invaded cells in each group. Data are presented as mean ± SD from three independent experiments. Student’s *t*-test was used for two-group comparisons, and one-way ANOVA followed by Tukey’s post hoc test was used for multiple-group comparisons. ** *p* < 0.01, *** *p* < 0.001.

qRT-PCR analysis showed that BIRC3, CCL20, and MMP9 mRNA levels were higher in suspension-cultured LUAD cells than in adherent-control cells, supporting their association with matrix-detachment stress in a cell-based model (Figure 7A). Because BIRC3 was prioritized by the computational model and candidate-compound screening, it was selected for further rescue analysis. In non-transfected LUAD cells, SB431542 treatment reduced BIRC3 and MMP9 protein levels and increased cleaved caspase-3, with more pronounced changes under the high-dose condition (Figure 7B,C). This pattern was consistent with reduced BIRC3/MMP9 expression and apoptotic activation after SB431542 treatment. Transwell assays further showed that SB431542 significantly decreased LUAD cell invasion, whereas BIRC3 overexpression partially restored the number of invaded cells under SB431542 treatment (Figure 7D,E). Together, these cell-based findings support a potential role of BIRC3 in SB431542-associated inhibition of LUAD cell invasion, whereas whether BIRC3 directly contributes to SB431542-induced apoptotic activation remains to be determined.

## 4. Discussion

In this study, we systematically investigated anoikis-related molecular features associated with brain metastasis in lung adenocarcinoma (LUAD) by integrating single-cell transcriptomics with machine learning-based analyses. Using the GSE131907 and GSE148071 datasets, we constructed a single-cell atlas encompassing both primary and brain-metastatic lesions. A methodological feature of this study was the use of a GPT-4 large language model (LLM)-assisted cell annotation protocol [28,29,30]. Compared with traditional methods such as SingleR or marker gene-based approaches, the LLM-assisted protocol provided complementary contextual information for annotating heterogeneous metastatic microenvironments, supporting the annotation of epithelial cells and immune subsets across distinct functional states [31]. This cell characterization provided a basis for subsequent quantitative analysis of anoikis-related activity and suggested that metastatic epithelial cells may acquire enhanced survival-related states during detachment and metastatic dissemination.

Cellular communication analysis suggested that enhanced anoikis-related activity may be associated with complex intercellular communication networks within the tumor microenvironment (TME) [32,33]. AUCell scoring showed significantly elevated anoikis scores in brain-metastatic cells, which was accompanied by enrichment of EMT-related processes and MYC target activity [34]. Notably, CellChat analysis highlighted the *SPP1* and *MIF* signaling axes as potentially relevant communication pathways. In the brain-metastatic microenvironment, macrophages frequently interacted with epithelial cells through the *MIF*-(*CD74*+*CXCR4*) and *SPP1*-*CD44* axes. These inferred interactions may be related to anti-apoptotic phenotypes in tumor cells and remodeling of the surrounding stromal microenvironment. This putative ‘metabolism-immune-survival’ interaction pattern may provide a biological explanation for enhanced survival and clonal expansion of LUAD cells in the brain parenchyma.

For bulk-transcriptomic modeling, we applied the LLM-enhanced integrative machine learning framework (LLM-IMLF) as an integrative strategy for prognostic signature construction [35]. This strategy aimed to construct an anoikis-related prognostic signature and further evaluate its exploratory ability to distinguish primary LUAD from brain-metastatic LUAD samples. Traditional bioinformatics models optimized for a single endpoint may show limited generalizability across independent datasets [36,37,38]. To improve model robustness, 101 machine learning algorithm combinations were compared for prognostic modeling, and the locked signature was subsequently assessed in independent prognostic and classification cohorts. The signature was developed in the TCGA prognostic training cohort, externally validated in GSE26939 for prognosis, and further evaluated in GSE161116 and GSE271259 for primary-versus-brain-metastatic LUAD classification. Through this prognosis-oriented screening and downstream classification assessment, the eight core signature genes (*BIRC3*, *CCL20*, *CLEC7A*, *CTSL*, *GOLM1*, *ICAM3*, *MTUS1*, and *SERPINH1*) showed consistent prognostic relevance and potential classification utility. Specifically, *BIRC3*, a core member of the inhibitor of apoptosis protein family, has been implicated in apoptosis regulation and may contribute to anoikis-related tumor cell survival through caspase-related pathways [39]. *CCL20* and *CLEC7A* may be involved in immune microenvironment remodeling in metastatic lesions, with the former associated with immunosuppressive cell recruitment and the latter related to myeloid receptor-mediated immune regulation [40,41]. *CTSL* and *SERPINH1* are related to extracellular matrix remodeling, invasive phenotypes, and stromal adaptation [42,43]. *GOLM1* and *ICAM3* may participate in vesicular transport, adhesion-related signaling, and metastatic adaptation [44,45,46]. Dysregulation of *MTUS1* has been associated with malignant migration and microtubule network dynamics [47]. This prognosis-derived anoikis-related signature captures biological processes related to apoptosis regulation, immune remodeling, and extracellular matrix reorganization, providing a potential tool for survival risk stratification and exploratory classification of primary versus brain-metastatic LUAD samples.

Finally, this study explored translational relevance through an exploratory path of ‘computational prediction–structural assessment’. CIBERSORT analysis suggested an association between high-risk scores and an immunosuppressive microenvironment, with high-risk patients showing features related to T-cell exhaustion and altered expression of immune checkpoints such as *TIGIT* and *CTLA4* [48,49], suggesting a potential link between anoikis-related risk and immune evasion [50]. For drug screening, we employed an LLM-assisted filtering step to prioritize candidate molecules, considering reported pharmacological properties and predicted blood–brain barrier (BBB)-related features. Molecular docking using AlphaFold-predicted target structures revealed that candidate drugs, including SB431542 and Resveratrol, bound favorably to the core targets [51,52]. Although these findings require further functional validation, the LLM-IMLF framework provides a systematic perspective from single-cell mechanisms to candidate compound prioritization and may support further mechanistic and translational studies in LUAD brain metastasis.

## 5. Conclusions

This study integrated single-cell transcriptomics with an LLM-assisted integrative machine learning framework to establish an eight-gene anoikis-related signature for prognostic assessment in LUAD. The signature also showed an exploratory ability to distinguish primary LUAD from brain-metastatic LUAD samples. Single-cell and immune microenvironment analyses revealed potential associations among anoikis-related activity, tumor–microenvironment interactions, and immune remodeling. Additionally, LLM-assisted compound prioritization combined with molecular docking identified several candidate compounds for hypothesis generation, which require further functional, pharmacokinetic, and in vivo validation.

## Figures and Tables

**Table 1:** Prognostic risk score of core genes.

Genes	Coef	HR	HR.95L	HR.95H	*p* Value
BIRC3	0.28811602	1.33391206	1.16768139	1.52380726	2.21 × 10^−5^
CCL20	0.12201213	1.12976781	1.04711984	1.21893908	0.00164467
CLEC7A	−0.3207868	0.72557793	0.61627277	0.85426999	0.0001178
CTSL	0.21752479	1.24299624	1.06593122	1.44947405	0.00553234
GOLM1	0.18613432	1.20458405	1.00363167	1.44577215	0.04561984
ICAM3	−0.7027885	0.49520252	0.33262848	0.73723552	0.00053733
MTUS1	−0.2356526	0.79005509	0.66243772	0.9422577	0.00875075
SERPINH1	0.32185661	1.37968693	1.11622553	1.70533283	0.00291128

## Data Availability

The transcriptomic datasets analyzed in this study are publicly available from the Gene Expression Omnibus (GEO) and The Cancer Genome Atlas (TCGA). Single-cell RNA-seq datasets (GSE131907 and GSE148071) and bulk RNA-seq datasets (GSE26939, GSE161116, and GSE271259) were obtained from GEO. The TCGA-LUAD dataset can be accessed via the GDC data portal. All data supporting the findings of this study are included in the article and Supplementary Information. Additional information is available from the corresponding author upon reasonable request.

## Supplementary Materials

The following supporting information can be downloaded at: https://www.mdpi.com/article/10.3390/cancers18121969/s1, Table S1. Sample information for primary and brain-metastatic LUAD single-cell RNA-seq datasets. Table S2. Sample information and survival outcomes for prognostic cohorts. Table S3. Sample information for primary versus brain-metastatic LUAD classification cohorts. Table S4. Workflow and model settings for LLM-assisted cell annotation. Table S5. Benchmarking performance of LLM-assisted and reference-based cell annotation methods. Table S6. Confusion matrix for LLM-assisted cell annotation. Table S7. Curated anoikis-related gene set used for scoring and candidate gene screening. Table S8. Performance matrix of 101 machine-learning algorithm combinations. Table S9. Primer sequences used for qRT-PCR analysis.
